# Rapunzel Syndrome: A Rare Postpartum Case

**DOI:** 10.1155/2013/857374

**Published:** 2013-09-19

**Authors:** Teshome Tegene, Yahia Foda, Omar Hussain, Kolawole Oloniyo, Ngoc-Tram Ha, Geeta Manikonda

**Affiliations:** ^1^Department of Internal Medicine, Prince George's Hospital Center, 3001 Hospital Drive, Cheverly, MD 20785, USA; ^2^Ross University School of Medicine, P.O. Box 266, Roseau, West Indies, Dominica

## Abstract

The Rapunzel syndrome describes a disorder in which a significant amount of hair is swallowed, forming a trichobezoar that extends past the stomach into the small intestines. Given the indigestible nature of hair, it subsequently leads to obstruction within the gastrointestinal system. Clinically, patients may present with symptoms of gastrointestinal obstruction, including abdominal complaints such as pain, nausea, vomiting, and diarrhea. However, due to its broad and nonspecific presenting symptoms, the diagnosis of Rapunzel syndrome warrants consideration once other common etiologies have been excluded. Surgical intervention is often required to remove the abdominal mass. This unusual syndrome is often associated with psychiatric disorders, affecting young women most commonly. In this report, we will discuss a unique case of Rapunzel syndrome in a one-month postpartum woman.

## 1. Introduction

The term “Rapunzel” originates from the well-known German fairy tale written by the Grimm Brothers in 1812, which tells the story of a 12-year-old girl Rapunzel who lived locked up inside a tower located in the middle of a forest [1]. Her beautiful long, golden hair is a defining symbol of this antiquated story. In medicine, a trichobezoar is defined as a collection of ingested hair located in the gastrointestinal tract that forms a compacted mass. Rapunzel syndrome is a rare and unusual variant in which a gastric trichobezoar extends past the duodenum and into the small bowel or beyond. First reported by Vaughan et al. in 1968, there only have been a few known reports published in the literature since [2]. Here, we will present yet another rare case of Rapunzel syndrome in a postpartum female. 

## 2. Case Report

 A four week postpartum 22 year old gravida 5 para 4 female with a past medical history of depression and iron deficiency anemia presented with a 2-week duration of progressive and constant, moderately severe epigastric pain. It radiated to the chest, increased with food intake, and was associated with nausea, nonbloody vomiting, and diarrhea. She denied any fever, chills, weight loss, abnormal vaginal discharge, chest pain, shortness of breath, eating out, recent travels, or exposure to sick contacts. She also denied any history of smoking, alcohol abuse, or illicit drug use. Her family history was only positive for hiatal hernia and diverticulosis. 

**Figure 1:**
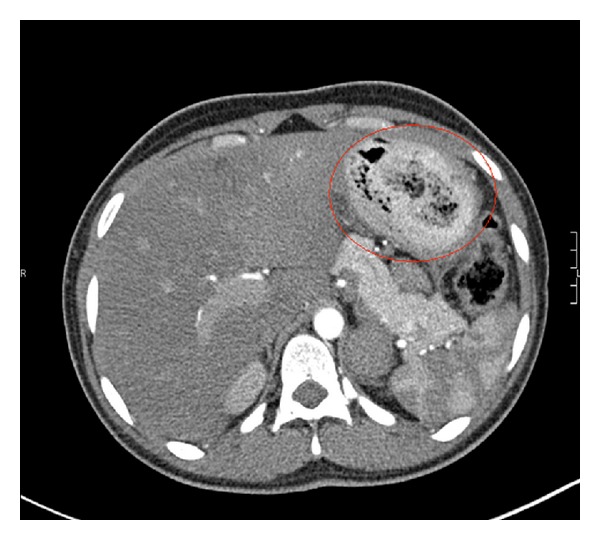
Computed tomography of the abdomen showing a trichobezoar located in the stomach (circled in red).

Her physical examination was completely unremarkable except for abdominal tenderness localized mainly to the left upper quadrant. On admission, her labs included white blood cell count 8500/*μ*L, hemoglobin 9.6 g/dL, hematocrit 30.7 g/dL, and platelet count 217,000/*μ*L. No electrolyte disturbances were noted. The remaining laboratory investigation showed iron 67 ng/mL, ferritin 17 ng/mL, TIBC 256, % Sat 26, TSH 0.437 *μ*IU/mL, B_12_ 747 pg/mL, folate 10.8 g/dL, and negative *β*-hCG. She was admitted to the hospital with a differential diagnosis of peptic ulcer disease, gastroesophageal reflux disease, and acute gastroenteritis. Computed tomography of the abdomen was ordered which showed an indistinct mass of material within the stomach (Figure 1) and a thickening of the duodenum and jejunum.

**Figure 2:**
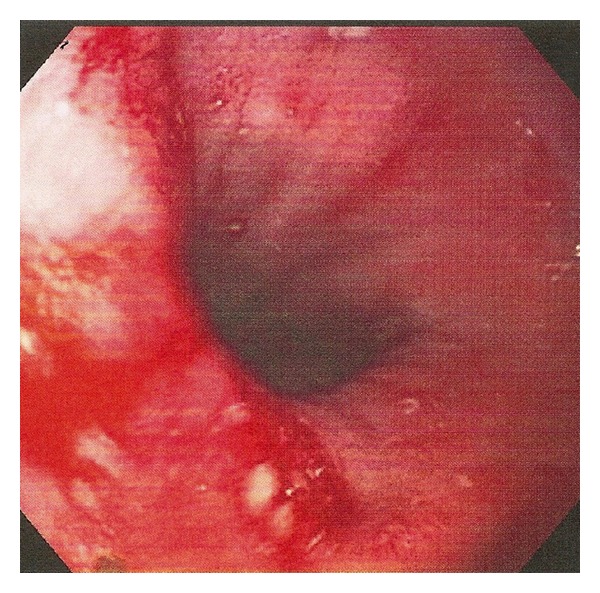
Hiatal hernia discovered during endoscopy.

**Figure 3:**
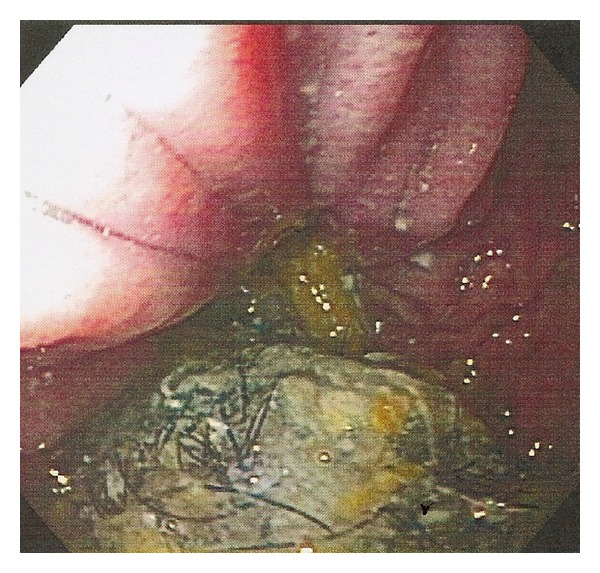
Endoscopic image of the gastric trichobezoar (greenish mass with dark strands of hair at the bottom of the image).

On the night of admission, the patient continued to have 3-4 episodes of nonbloody vomiting. Esophagogastroduodenoscopy (EGD) was scheduled for the following morning to further investigate the etiology of her gastrointestinal symptoms. EGD revealed the presence of a hiatus hernia (Figure 2) and a gastric foreign body identified as a trichobezoar (Figure 3). Subsequent endoscopic removal of the mass was attempted but was unsuccessful. 

**Figure 4:**
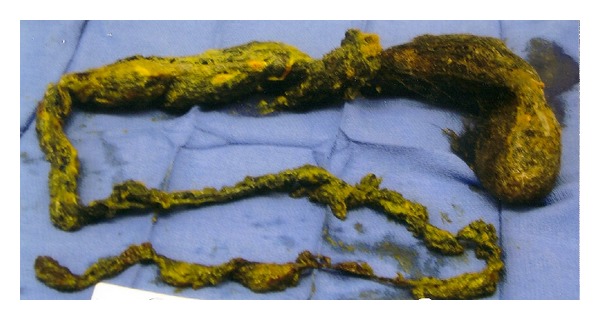
A 100 cm long gastroduodenal trichobezoar that was removed by gastrotomy. The larger portion at the top right corner of the image was located in the stomach. Notice how the mass decreased in width as it extended further down into the small intestines.

Upon further questioning, she admitted to having abnormal cravings for hair during the last 3 months of her most recent pregnancy. More specifically, she reported that she had been ingesting excessive amounts of hair from her wigs during the last trimester of pregnancy, causing her to change wigs every 2-weeks. Interestingly, she also reported having consumed the threading of washcloths throughout one of her other previous pregnancies, but denied experiencing any gastrointestinal symptoms associated with those particular cravings or any hospitalization at that time. There was no desire to eat the aforementioned nonfood items outside of the times of pregnancy. A psychiatric assessment was performed and an Axis I diagnosis of anxiety, not otherwise specified, was made. Surgery was scheduled after endoscopic discovery of the intragastric mass. Gastrotomy was performed and a gigantic 100 cm long gastroduodenal trichobezoar was extracted, extending down to the jejunum (Figure 4). No bowel perforation or other intestinal pathology was noted during the procedure. Surgery was uncomplicated and the patient remained stable post operatively during the remainder of her hospital course through discharge. Discharge recommendations included further psychiatric counseling. She was contacted several months later for follow up and denied having any additional unusual cravings. 

## 3. Discussion

Trichobezoars are accumulations of hair or hair-like material within the gastrointestinal tract [3]. In most cases, trichobezoars are confined within the stomach, but sometimes they may extend through the pylorus into the jejunum, ileum, and on rare occasions even the colon. It is well established in the literature that the Rapunzel syndrome mainly occurs in young female patients with psychiatric disorders [4, 5]. To date, there have been less than 40 cases of Rapunzel syndrome documented. Our patient displays a rather unique case of this rare syndrome since these individuals only rarely eat hair from other sources such as wigs [3]. Although the prevalence rate of bezoars in humans is low, if left untreated, the mortality rate can rise as high as 30% because of complications, including gastrointestinal destruction, bleeding, intussusception, or perforation [6]. Death, fortunately, is a rare complication [7, 8]. 

 Bezoars are often times incidental findings in a work up of a patient presenting with nonspecific symptoms. Upper gastrointestinal tract obstruction is the most common presentation of a gastric trichobezoar [9]. If a patient is suspected of having a trichobezoar, a thorough history and physical exam should be performed in order to correctly arrive at the diagnosis. Questioning should include any history of ingesting nonfood products in the past. Particularly in pregnant or postpartum females, one should inquire about strange eating habits during the current and prior pregnancies in order to aid in a suspected diagnosis of trichobezoar. In fact, trichobezoar may be associated with pregnancy and iron deficiency anemia, which often results in a syndrome known as pica. In a prospective study with 227 adult pregnant women, pica was diagnosed in 14.4% out of whom almost half practiced it daily. The onset occurred in the second trimester in 46.7% of the cases and 30% experienced pica during their third trimester [10]. 

On physical examination, thorough palpation of the abdomen is needed so that the diagnosis of trichobezoar is not missed. Patchy alopecia and severe halitosis can also be noted [3]. Further investigation can be done using imaging modalities such as abdominal radiographs with or without barium, abdominal ultrasound, or CT scan. The bezoar can be seen as a mass or filling defect. CT scan may have higher accuracy in comparison to ultrasound when attempting to locate the mass. However, upper endoscopy remains the gold standard for diagnosis as it provides direct visualization of the gastric mass, allows sample taking, and serves as a therapeutic intervention simultaneously. Endoscopic removal can be attempted if the bezoar is confined to the stomach. However, in the case of Rapunzel syndrome, surgical gastrotomy is oftentimes recommended because of the extent of gastrointestinal involvement by the bezoar. Infection, bleeding, and leakage of gastric contents from the suture line are problems that may occur following surgical intervention. 

## 4. Conclusion

Rapunzel syndrome is an uncommon disorder, which frequently can be missed initially given its nonspecific presentation and low incidence rate. After the exclusion of more common etiologies, trichobezoar should be considered in all young females presenting with symptoms of gastrointestinal obstruction, including nausea, vomiting, abdominal pain, and diarrhea. A thorough physical examination and history should be obtained to support the clinical suspicion. In addition, abdominal computed tomography and upper endoscopy should be included in the work up to ensure appropriate diagnosis and intervention in order to avoid fatal complications such as intussusception and perforation. Lastly, a proper psychiatric evaluation and follow-up should be performed to prevent any recurrences.
